# Expert opinion on monitoring symptomatic hereditary transthyretin-mediated amyloidosis and assessment of disease progression

**DOI:** 10.1186/s13023-021-01960-9

**Published:** 2021-10-03

**Authors:** David Adams, Vincent Algalarrondo, Michael Polydefkis, Nitasha Sarswat, Michel S. Slama, Jose Nativi-Nicolau

**Affiliations:** 1 Université Paris-Saclay, U1195, INSERM, Le Kremlin Bicêtre, France; 2 Neurology Department, AP-HP, CHU Bicêtre, Le Kremlin Bicêtre, France; 3 Cardiology Department, CHU Bichat-Claude-Bernard, 46 rue Henri Huchard, 75018 Paris, France; 4 Department of Neurology, Johns Hopkins Hospital, 855 North Wolfe Street, Baltimore, MD 21205 USA; 5 Department of Medicine, University of Chicago, 5841 S Maryland Ave, Chicago, IL 60637 USA; 6 Department of Internal Medicine, University of Utah, 30 N 1900 E, Salt Lake City, UT 84132 USA

**Keywords:** hATTR amyloidosis, ATTRv amyloidosis, Amyloid neuropathies, Amyloid cardiomyopathies, Familial, Diagnostic techniques and procedures, Disease progression, Transthyretin

## Abstract

**Background:**

Hereditary transthyretin-mediated amyloidosis, also known as ATTRv amyloidosis (v for variant), is a rare, autosomal dominant, fatal disease, in which systemic amyloid progressively impairs multiple organs, leading to disability and death. The recent approval of disease-modifying therapies offers the hope of stabilization or eventual reversal of disease progression, and yet highlights a lack of disease-management guidance. A multidisciplinary panel of expert clinicians from France and the US came to consensus on monitoring the disease and identifying progression through a clinical opinion questionnaire, a roundtable meeting, and multiple rounds of feedback.

**Monitoring disease and progression:**

A multidisciplinary team should monitor ATTRv amyloidosis disease course by assessing potential target organs at baseline and during follow-up for signs and symptoms of somatic and autonomic neuropathy, cardiac dysfunction and restrictive cardiomyopathy, and other manifestations. Variability in penetrance, symptoms, and course of ATTRv amyloidosis requires that all patients, regardless of variant status, undergo regular and standardized assessment in all these categories. Progression in ATTRv amyloidosis may be indicated by: worsening of several existing quantifiable symptoms or signs; the appearance of a new symptom; or the worsening of a single symptom that results in a meaningful functional impairment.

**Conclusions:**

We suggest that a multisystem approach to monitoring the signs and symptoms of ATTRv amyloidosis best captures the course of the disease. We hope this work will help form the basis of further, consensus-based guidance for the treatment of ATTRv amyloidosis.

**Supplementary Information:**

The online version contains supplementary material available at 10.1186/s13023-021-01960-9.

## Background

### Disease definition

Hereditary transthyretin-mediated amyloidosis, also known as ATTRv (v for variant) amyloidosis, is a rare, progressively debilitating, and fatal systemic disease caused by pathogenic variants in the transthyretin (*TTR*) gene [1–4]. Primarily synthesized by the liver [5–7], TTR circulates as a tetramer involved in the transport of the vitamin A–retinol binding protein complex and plays a minor role in thyroxine transport [8, 9]. However, in the case of ATTRv amyloidosis, pathogenic *TTR* variants result in synthesis of unstable TTR tetramers, which then dissociate into monomers, misfold, and aggregate into amyloid fibrils [10, 11]. These fibrils subsequently accumulate in the extracellular spaces of multiple (mostly non-mitotic) organs and tissues, such as the nerves, heart, eyes, and gastrointestinal (GI) tract [1, 3]. The result is a multisystem disease, which can manifest with intractable somatic and autonomic neuropathy, and/or cardiomyopathy, in addition to other disease signs and symptoms [2, 3, 12–14] (Table 1).

**Table 1:** Symptomatology of ATTRv amyloidosis

Impairment	Site of amyloid deposition	Associated symptoms or conditions
Bilateral sensorimotor polyneuropathy [1, 3]	Somatic nerve fibers	Neuropathic pain or numbness in hands and feet
		Walking difficulties, balance disorders
		Loss of grip strength
Autonomic dysfunction [14, 15]	Autonomic nerve fibers	Sexual dysfunction
		Disturbances in GI motility
		Urinary disorders
		Sweating abnormalities detected by clinical tests
		Eye dryness
Infiltrative cardiomyopathy [16–20]	Cardiac extracellular matrix	Dyspnea, peripheral edema
		Decrease of performance/6-min walk test
		Syncope
		Fatigue
		Bradyarrhythmias or tachyarrhythmias
Cardiac dysautonomia [14, 21–23]	Autonomic cardiac nerves	Lack of increase in heart rate during exercise
		Orthostatic hypotension
		Syncope
Ophthalmic impairment [15, 22]	Eye	Blurred vision
		Vitreous opacities
		Glaucoma
Connective tissue manifestations [15, 24–27]	Tenosynovial tissues, ligaments, tendons	Carpal tunnel syndrome

*ATTRv* hereditary transthyretin (v for variant), *GI* gastrointestinal

Transmission of ATTRv amyloidosis is autosomal dominant and multiple factors may affect time of onset and disease natural history [4]. Over 120 pathogenic *TTR* variants have been identified [28], with prevalence of different mutations varying by geography. The most common *TTR* mutation in Europe is V30M (V50M) [29], with prevalence reaching up to 1 in 1000 in endemic areas in Portugal, Sweden, and Japan [3, 29]. In comparison, the most common *TTR* mutation in the US is V122I (V142I), which has a reported prevalence of approximately 4% in African Americans [17]. There is a notable genotype–phenotype variability, and some specific *TTR* mutations have historically been associated with particular disease manifestations, with V30M most often associated with predominant polyneuropathy and V122I most often associated with predominant cardiomyopathy [30]. However, progress in understanding the disease, and careful clinical and imaging observation, has led to recognition that there is frequent multisystem involvement across all *TTR* variants [31], and a mixed phenotype including polyneuropathy and cardiomyopathy can be found in the majority of patients with ATTRv amyloidosis [30–33]. Furthermore, there is considerable variability in the penetrance, symptoms, and course of the disease across *TTR* variants [34, 35], even within families [36]. This variability and lack of specificity of symptoms, allied to low disease awareness and incomplete penetrance of ATTRv amyloidosis, presents a challenge for diagnosis of symptomatic patients [37–39].

### Natural history and mortality

The age of disease onset and rate of progression of ATTRv amyloidosis can differ among various target organs, yet, overall, once symptoms appear the disease advances rapidly without treatment. This disease progression is associated with increasingly severe symptomatology, disability, and mortality [1]. Among patients with ATTRv amyloidosis with predominant polyneuropathy, disease progression manifests with the extension of sensory loss from the feet to proximal lower limbs, onset of weakness in lower limbs extending later to hands, and autonomic dysfunction of increasing severity. The rapidity of worsening is increased in late-onset (LO, onset > 50 years) disease compared with early-onset (EO) disease. The impact on locomotion is progressive and major, with patients requiring assistance with walking within 3–5 years from onset, and being wheelchair-bound within 5–10 years, depending on the age of onset and *TTR* variant. The multifaceted impairment related to ATTRv amyloidosis is associated with poor prognosis, with the median overall survival following diagnosis reported to be 4.7 years [40]. Survival in patients presenting with predominant cardiomyopathy is further reduced to 3.4 years [40, 41], with death usually due to progressive heart failure (HF) or to life-threatening cardiac arrhythmia [42, 43]. Survival from disease onset in patients with ATTRv amyloidosis with polyneuropathy ranges from approximately 12 years in the EO V30M variant to approximately 7 years in other variants such as LO V30M and I107V [44, 45]. The variation in survival shown in Additional file 1: Table S1 (see Additional file 1) highlights the shorter survival seen in patients with variants associated with predominant cardiomyopathy.

### Unmet need in disease monitoring and progression

Disease-modifying therapies (DMTs) for ATTRv amyloidosis have evolved considerably over the past 30 years [4]. From the 1990s to the early 2010s, orthotopic liver transplantation was the only treatment strategy available that targeted the pathogenic variant TTR protein [15, 46], yet a range of disease-modifying pharmacotherapies are now available including small-molecule TTR stabilizers such as tafamidis and diflunisal, and gene-silencing drugs such as patisiran and inotersen [32, 33, 47–49].

To address the challenges around choice and initiation of these DMTs, careful monitoring of the multiple potential signs of disease progression is required. While some guidance exists for monitoring presymptomatic individuals [50, 51], there is limited consensus on monitoring symptomatic individuals [52], and progression remains poorly defined. Here, it is likely important to utilize multiple measures at baseline and during follow-up assessments, as often these can detect progression before clear clinical worsening.

The authors, a panel with expertise in ATTR amyloidosis comprising a neurologist and 2 cardiologists from France and a neurologist and 2 cardiologists from the US, answered a questionnaire on monitoring disease state and detecting disease progression based on a literature review of expert opinion articles and management guidelines on ATTRv amyloidosis. At a virtual roundtable meeting in July 2019, the author team met to discuss the pooled questionnaire results. A set of recommendations on monitoring and disease progression were formulated using the team’s experience of treating patients with ATTRv amyloidosis, their knowledge of the literature, and an analysis of clinical trials. These were further developed over 4 rounds of feedback.

The recommendations have 3 aims. First, to identify the signs, symptoms, and tests with which to monitor patients with ATTRv amyloidosis. Second, to define changes in the identified measures that signify meaningful disease progression and thereby identify non-responders. Finally, to specify the frequency of disease-monitoring assessments required for timely identification of progression.

## Disease monitoring

### Baseline assessment

Patient monitoring should start immediately after diagnosis to provide a baseline assessment from which to judge the disease course. Ideally, assessments should take place at a specialist center with experience of ATTRv amyloidosis and utilize a multidisciplinary team that reflects the multisystemic nature of this disease. At minimum, this team should comprise a neurologist and a cardiologist, a genetic counselor, and an ophthalmologist. Other specialties such as gastroenterologists, nutritionists, physical therapists, nephrologists, and urologists, should be consulted as needed.

Members of the multidisciplinary team should strive to capture the full range of disease manifestations by assessing somatic and autonomic neuropathy, cardiac dysfunction, and other disease manifestations. Particular attention should be paid to using a quantified approach when possible, as clinical signs and symptoms can vary from patient to patient. Proposed signs and symptoms to be assessed are shown in Table 2.

**Table 2:** Monitoring signs and symptoms in patients with ATTRv amyloidosis

Area of impairment	Subjective symptoms		Objective signs	
	Symptom	Assessments (questionnaire)	Signs	Assessments
Somatic neuropathy	Pain, paresthesia	VAS (0–10)	Sensory loss for pain in LLs and ULs	Extension of sensory loss in LLs and ULs
		Extension on the body		NCS
				Skin punch biopsy
	Walking difficulties	Walking perimeter	Walking difficulties	10MWT
		PND score (0–IV)		Timed Get Up and Go test
				NIS (0–192)
	Balance disorders	Falls	Balance disorders	Romberg sign
				Apallesthesia in the feet
	Disability, difficulties with fine gestures	R-ODS (48–0)	Weakness in all 4 limbs	NIS
				Grip test
Autonomic neuropathy	Faintness, syncope	Questionnaire	Cardiovascular dysautonomia	HRV test
				MIBG cardiac scintigraphy
				Atropine IV test
			Orthostatic hypotension	Serial supine and orthostatic BP and pulse
	Diarrhea, constipation, alternating diarrhea–constipation, early satiety, vomiting		Early satiety	Weight
				mBMI
			Gastroparesis	Gastric emptying test
	Sweating abnormalities		Sweating abnormalities	Sudoscan®
	Urinary retention, incontinence, sexual dysfunction	Questionnaire		Urodynamic assessment
	Overall autonomic symptoms	COMPASS-31 (0–100)		
		CADT (20–0)		
Amyloid cardiomyopathy	Excessive exertional tachycardia/syncope/bradycardia/palpitations (or none in early disease)	Questionnaire	Cardiac arrhythmia/atrial fibrillation	ECG
			Conduction disorders	24-h Holter ECG
				EPS
	Shortness of breath, fatigue, weight gain, fluid retention in lower extremities, abdominal swelling	Questionnaire	Heart failure	Clinical examination including auscultation of heart and lungs
		NYHA class	Volume overload/jugular venous distension/gallop rhythm/crackles (crepitant rales)/lower extremity edema	Body weight increase
				6MWT
				NT-proBNP
				Troponin
			Cardiac imaging	Echocardiogram
				cMRI
				DPD/PYP scintigraphy
Ocular manifestations	Ocular symptoms (blurred vision)	Questionnaire	Ocular dysautonomia	ACVs
				KCS
				Pupillary abnormalities
			Amyloid deposition	Glaucoma (tonometry)
				Vitreous opacities (slit lamp examination)
Renal dysfunction	Fatigue, decreased urine output	Questionnaire	Renal dysfunction	eGFR
				Albuminuria
				Urine proteinuria
General health	Fatigue	Questionnaire		
	Cachexia	Body weight decrease	General health	mBMI
	Quality of life	Norfolk QOL-DN questionnaire (− 4 to 136)		BMI
				Prealbumin
		SF-36 questionnaire (0–100 per scale)		
		KCCQ^a^ (100–0)		

*6MWT* 6-min walk test, *10MWT* 10-m walk test, *ACV* abnormal conjunctival vessel, *ATTRv* hereditary transthyretin (v for variant), *BMI* body mass index, *BP* blood pressure, *CADT* Compound Autonomic Dysfunction Test, *cMRI* cardiac magnetic resonance imaging, *COMPASS-31* Composite Autonomic Symptom Score-31, *DPD* ^99m^Tc-3,3-diphosphono-1,2-propanodicarboxylicacid, *ECG* electrocardiogram, *eGFR* estimated glomerular filtration rate, *EPS* electrophysiologic study, *HRV* heart rate variability, *IV* intravenous, *KCCQ* Kansas City Cardiac Questionnaire, *KCS* keratoconjunctivitis sicca, *LL* lower limb, *mBMI* modified body mass index, *MIBG* metaiodobenzylguanidine, *NCS* nerve conduction study, *NIS* neuropathy impairment score, *Norfolk QOL-DN* Norfolk Quality of Life-Diabetic Neuropathy, *NT-proBNP N*-terminal prohormone of brain-type natriuretic peptide, *NYHA* New York Heart Association, *PND* polyneuropathy disability, *PYP* ^99m^Tc-pyrophosphate, *R-ODS* Rasch-built Overall Disability Scale, *SF-36* 36-item Short-Form Health Survey, *UL* upper limb, *VAS* visual analog scale

^a^KCCQ is specific to patients with cardiac disease

### Somatic neuropathy

Most patients presenting with neuropathy have both sensory and motor symptoms. Early motor involvement can often distinguish ATTRv amyloidosis from sensory-predominant neuropathies such as those associated with diabetes. Additionally, ATTRv amyloidosis can often be associated with early hand involvement due to focal neuropathies such as carpal tunnel syndrome. Common somatic neuropathy symptoms include pain, paresthesia, walking difficulties, balance disorders, and difficulties with fine dexterity. On examination, signs include extent of sensory loss for pain in the lower limbs, areflexia, apallesthesia in the feet, and weakness (Table 2).

A clinical assessment should cover familial amyloid polyneuropathy (FAP) stage [53] and/or polyneuropathy disability (PND) score [54] by interview, and the neuropathy impairment score (NIS) [55] by examination. FAP stage and PND score assess disability with a focus on impairment of ambulation [53, 54, 56]. A simple NIS should be captured at baseline, to reflect the severity of the somatic neuropathy [1], although a range of different composite NIS tools are available [57, 58]. These composite measures have been used in clinical trials as they have evolved for use in ATTRv amyloidosis, although in their full iteration they are often considered too complex and time-consuming for routine clinical purposes. It should also be noted that the NIS, like many other scales used in the assessment of ATTRv amyloidosis, is not a linear scale so the impact of a specific score change may differ according to the patient’s starting level of neuropathy impairment. A simpler clinical examination can include the 10-m walk test [59] or the Timed Get Up and Go test [60], which can be used to assess gait and balance, even in patients with disabling neuropathy. Other relevant neurologic tests include the Jamar Hydraulic Hand Dynamometer (Sammons Preston Rolyan, Bolingbrook, IL, US) [1] both hands grip strength test, which can be performed in consultations, nerve conduction studies, skin punch biopsies, and quantitative sensory testing using CASE IV (WR Medical Electronics Co, Maplewood, MN, US).

Patient disability assessment by the Rasch-built Overall Disability Scale (R-ODS) [61] questionnaire is also of notable value, since this is tailored to patients with peripheral neuropathy and measures the effect on activities of daily living [61]. Scores on this scale can capture functional impairment by assessing the ability of an individual to function independently in daily life. R-ODS score should be assessed before each consultation.

### Autonomic neuropathy

Autonomic neuropathy affects several organs and its signs and symptoms can be some of the earliest manifestations of ATTRv amyloidosis. Despite this, the extent of autonomic neuropathy can be difficult to assess due to the large variety of possible symptoms that may be related to this condition and the relatively few empirical signs that can be used to measure progression (Table 2).

Autonomic dysfunction can manifest with a range of symptoms, including GI (e.g. early satiety, chronic diarrhea, constipation, and gastroparesis) and genitourinary (e.g. sexual dysfunction, urinary retention, and urinary incontinence) problems which should all be monitored (Table 2). Sexual dysfunction and constipation are typically early symptoms, with sexual dysfunction often apparent within the first 2 years of onset of symptomatic disease. Conversely, urinary retention and urinary incontinence typically manifest in the later stages of the disease. Overall, the Compound Autonomic Dysfunction Test [62], integrating evaluation of postural hypotension, nausea/vomiting, diarrhea/constipation, and sphincter disturbances, is a simple and reproducible scale which is adapted to evaluate the main symptoms of autonomic dysfunction observed in ATTRv amyloidosis with polyneuropathy [62]. Specific signs of autonomic neuropathy include skin conductance, which can be assessed for sudomotor function by Sudoscan^®^ [63, 64], and autonomic and sensory symptoms, which can be captured by the small-fiber neuropathy and symptom inventory questionnaire.

Cardiac dysautonomia involves both sympathetic and parasympathetic systems, and/or the balance between them (Table 2). Signs of cardiac dysautonomia can be detected by: assessment of orthostatic hypotension (asymptomatic or resulting in faintness or syncope) using lying/standing measurement or tilt table test; heart rate variability with deep breathing test; heart rate variability (standard deviation [SD] of normal-to-normal R–R interval variability on a 24-h electrocardiogram [ECG] Holter recording); or the Valsalva maneuver [65, 66]. The parasympathetic system can be tested with the heart rate response to atropine intravenous infusion [67], and the sympathetic system by metaiodobenzylguanidine cardiac scintigraphy [68]. As with other signs and symptoms of autonomic neuropathy in ATTRv amyloidosis, tests for cardiac dysautonomia have a powerful early diagnostic and prognostic value, but are still rarely used [21, 22].

The Composite Autonomic Symptom Score-31 (COMPASS-31) questionnaire [60] is recommended for broad assessment of the severity and extent of a range of autonomic symptoms, including vasomotor, secretomotor, GI, and bladder dysfunction [69]. Although COMPASS-31 is not commonly used in a clinical setting, it has been used successfully in clinical trials to measure longitudinal changes in dysautonomia [23, 32] and could be collected before consultations.

### Amyloid cardiomyopathy

There are no specific signs of ATTR amyloid cardiomyopathy. Typical age of onset is > 50 years old, but variations can appear within a given family, so patients should be warned to seek medical advice in the event of any potential cardiac sign. Patients should be more closely monitored within 10 years of the typical age of onset in their family.

When monitoring cardiomyopathy, symptoms to be aware of include exertional dyspnea, volume overload, jugular distension, lower limb edema, cachexia, fatigue, weight increase, abdominal swelling, excessive exertional tachycardia, syncope, bradycardia, and palpitations (Table 2). History of recent unplanned cardiac hospitalization should also be recorded and taken into account.

All patients should undergo ECG, assessment of cardiac biomarkers [70] (*N*-terminal prohormone of brain-type natriuretic peptide [NT-proBNP], troponin I or troponin T, or high-sensitivity troponin), and functional exercise-based tests, such as the 6-min walk test (6MWT) [71]. However, interpretation of exercise-based tests, such as 6MWT can be complicated by concurrent peripheral neuropathy. Similarly, New York Heart Association (NYHA) classification, which is routinely used to assess the patient condition, has limited reproducibility and meaning in patients with peripheral neuropathy [72]. Cardiopulmonary exercise testing could be used as a second-line tool to quantify exercise tolerance in terms of blood pressure and heart rate response, and measure gas exchange to quantify the severity of HF; currently it is not routinely used for monitoring. However, it provides prognostic information in patients who have cardiac deterioration and are being considered for heart transplantation.

Multimodal cardiac imaging [73] (including echocardiography, “bone” scintigraphy [74, 75], and cardiac magnetic resonance imaging [cMRI] [76, 77]) is useful in monitoring the disease, particularly at crucial junctures. For example, this multimodal approach can be valuable at the patient's first evaluation or after significant progression has been detected using other assessments. For more regular patient monitoring, when there is no clear change in disease status, it is possible to just include a single imaging modality, such as echocardiography.

Typical echocardiography is that of preserved ejection fraction with a reduced left ventricular (LV) chamber and thickened myocardial walls. The thickened interatrial septum, thickened valves, and progressive dilatation of the left atrium are typically accompanied by elevation of LV filling pressure and pulmonary hypertension, while ejection fraction remains normal and end diastolic LV volume is normal or reduced. It is informative to obtain echocardiographic strain measurements, as this pathology is associated with reduced LV global longitudinal strain with apical sparing (typical apical-to-basal strain ratio > 2.1) with LV ejection fraction-to-strain ratio > 4. In the late stage of the disease systolic function is also impaired. cMRI typically shows thickened myocardium and morphologic features similar to those provided by echocardiogram, and may also show late gadolinium enhancement in ventricles and atria, increased values of longitudinal relaxation time (T1) mapping, and increased extracellular volume. Finally, cardiac scintigraphy shows uptake of the tracers used for bone scintigraphy (^99m^Tc-3,3-diphosphono-1,2-propanodicarboxylicacid or pyrophosphate) (which is never observed in a normal heart) in the setting of ATTR amyloidosis or light-chain amyloidosis. As such, it may be preferable to perform scintigraphy after gammopathy has been excluded by serum and protein electrophoresis or immunofixation electrophoresis. Single photon emission computed tomography of “bone” tracers allows quantification and reclassification of cardiac uptake grading in patients with ambiguous results on conventional planar acquisitions.

For the cardiac imaging techniques of echocardiography, “bone” scintigraphy, and cMRI, we recommend that, as far as possible, each type of assessment is performed by the same operator with experience of amyloid cardiomyopathy, on the same machine, using the same software, in order to ensure consistency and enable the tracking of small changes in measurements. The use of “cut off values” can be misleading, as they do not take into account the existence of a “gray zone” corresponding to disease onset. The limited reproducibility of cardiac imaging should also be taken into account during monitoring. For this reason, it is important that multiple assessments are taken, and that disease progression is not over-diagnosed based on changes in a single imaging modality.

Vigilance should be maintained regarding symptomatic or asymptomatic conduction abnormalities such as new bundle branch block or sinus node dysfunction, atrioventricular block (which may require electrophysiologic testing and/or pacemaker implantation), and cardiac arrhythmias (usually atrial fibrillation). An implantable cardiac monitor may assess the arrhythmia burden and identify the need for pacemakers or other treatments. If the patient already has a pacemaker implanted, interrogating the pacemaker memory can allow detection of bouts of asymptomatic atrial fibrillation requiring anticoagulation. Electrophysiologic study could also be considered in cases of asymptomatic conduction abnormalities, such as left or right bundle branch block and/or prolonged PR interval, or in cases of infiltrative cardiomyopathy, even if the ECG is normal [22].

In conjunction with these tests, it may also be useful to monitor patients using a cardiac amyloidosis-specific prognostic staging system. Grogan et al. described a system that classified patient risk based on threshold levels of 2 cardiac biomarkers (troponin T and NT-proBNP) in patients with amyloidogenic transthyretin (wild-type) (ATTRwt) amyloidosis [70]. Gillmore et al. described a similar system based on thresholds of NT-proBNP and estimated glomerular filtration rate (eGFR) in patients with either ATTRwt or ATTRv amyloidosis [78]. The staging systems predict increased risk of mortality in patients with levels of cardiac biomarkers above threshold and/or eGFR below threshold compared with patients not meeting those criteria [70, 78]. Recently, a retrospective study of 945 patients with ATTR amyloidosis with cardiomyopathy showed that baseline evaluation and progression of ATTR stage could predict mortality at follow-up [79]. However, their routine use in patient monitoring is not yet established worldwide.

Right-heart catheterization is clinically useful in patients undergoing evaluation for heart transplantation but is invasive in nature and thus considered unsuitable for routine monitoring. It is commonly replaced by refined echocardiographic assessment and use of NT-proBNP for a more physiologic hemodynamic assessment.

### Ophthalmologic manifestations

Ophthalmologic dysfunction caused by amyloid deposits in the vitreous body can be captured by testing for vitreous opacities and glaucoma whereas ocular autonomic dysfunction can be identified by testing for abnormal conjunctival vessels (ACVs), keratoconjunctivitis sicca (KCS), and pupillary abnormalities [80–83].

### Other disease manifestations and quality of life

Symptoms and signs caused by dysfunction in the renal systems are also shown in Table 2. Relevant renal tests include eGFR, creatinine clearance, albuminuria, and proteinuria.

Multiple organs and systems affected by ATTRv amyloidosis can cause a range of symptoms including unexplained weight loss, nausea, and fatigue. Reduction in quality of life (QOL) due to this multisystem impairment has been measured in clinical trials using the Norfolk Quality of Life-Diabetic Neuropathy (Norfolk QOL-DN) Questionnaire, 36-item Short-Form Health Survey (SF-36), and Kansas City Cardiac Questionnaire (KCCQ) [32, 33, 48, 84]. Although these questionnaires are not typically used in clinical practice, with suitable training they are easy to administer and can even be completed outside the clinic. Patient health more generally should be assessed by measuring body mass index (BMI), modified BMI (mBMI; BMI [kg/m^2^] × serum albumin [g/L]), and weight. mBMI is preferred to measure nutritional status in patients with ATTRv amyloidosis as low serum albumin levels and fluid retention may result in normal BMI measurements despite worsening nutritional status [85]. In patients receiving *TTR* gene-silencing therapies, it is also useful to monitor serum prealbumin at baseline and every 6 months.

## Disease progression

As ATTRv amyloidosis progresses, affected organs become increasingly impaired, and most of the associated symptoms become worse over the course of the disease [1]. Patient-based assessments must be considered, and the patient should be followed by the same physician to minimize subjective bias. Consequently, disease progression can be defined as:

- The worsening of several existing quantifiable symptoms, signs, or objective test results (Tables 2, 3, 4). For example, increased weight and dyspnea requiring increase of diuretics dose.
- The appearance of a new symptom.
- The worsening of a single symptom that results in a meaningful increase in functional impairment. For example, sensory loss in the fingertips which precludes a patient from dressing, or cooking for themselves, or interferes with their job.
- Several specific combinations of signs and symptoms that can indicate progression (Table 5).

**Table 3:** Assessments for monitoring progression in somatic and autonomic neuropathy in recommended order of importance

Assessment	Indicator of progression	Frequency of assessment	Sensitivity to progression^a^
Somatic neuropathy			
10MWT	Change in gait speed 0.05–0.10 m/s [93]	6–12 months	High
OR			
Timed Get Up and Go test	An increase of 15% over 6 months (or 30% over 12 months) in the time taken to stand up, walk across the room, and sit down	6–12 months	High
PND score	Change in disease stage	6–12 months	Low in EO V30M
	Not sensitive to small changes in progression but useful to assess during monitoring visits as a change in score indicates increased functional impairment		High in LO V30M
Jamar Hand Dynamometer—both hands grip strength test	Reduction of grip strength of 4–6 kilos over 12 months		
	In the APOLLO trial, least squares mean grip strength decreased by 43% over 18 months in patients treated with placebo (*n* = 56, personal communication)	6–12 months	High
R-ODS^b^	Worsening of R-ODS score by 3–8 points over 12 months or worsening of the score on 2 consecutive consultations 6 months apart (questionnaire to be filled in before the consultation)	6–12 months	High
SFN-SIQ	Particularly useful for monitoring patients with V30M and EO disease	6–12 months	Medium
Walking perimeter/balance disorders	Onset of balance disorders	6–12 months	High
	Falls		
	Reduction of walking perimeter in daily life (in meters)	6–12 months	Medium
NIS^b^	A change of 7–16 points over 12 months or worsening of the score on 2 consecutive consultations 6 months apart	6–12 months	High in LO V30M
	Give more weight to changes in strength and less weight to changes in reflexes		
NCS	Decrease of 20% amplitude in several nerves over 12 months when the same nerves are tested using the same methods over time	12 months at most	Medium
Autonomic neuropathy			
Sudomotor testing	Using Sudoscan^®^, a reduction on 2 consecutive examinations of the feet	12 months	High
	Rarely performed but useful for monitoring patients with V30M and EO disease		
Heart rate deep breathing	A change from an age-adjusted normal value to abnormal value	12 months	High
CADT questionnaire^b^	Reduction of total CADT score by 2 points or reduction of any subscore by 1 point	6 months	Low
OR			
COMPASS-31 questionnaire^b^	Increase by 1 point in a year	12 months	Low
Orthostatic vital signs	New onset of orthostatic hypotension	6 months	Medium
	Onset of orthostatic syncope for patients who already have orthostatic hypotension		
	Typically manifests in later stages of disease		
Valsalva maneuver	A change from an age-adjusted normal value to abnormal value	12 months	High

*10MWT* 10-m walk test, *CADT* Compound Autonomic Dysfunction Test, *COMPASS-31* Composite Autonomic Symptom Score-31, *EO* early-onset, *LO* late-onset, *NCS* nerve conduction study, *NIS* neuropathy impairment score, *PND* polyneuropathy disability, *R-ODS* Rasch-built Overall Disability Scale, *SFN-SIQ* small-fiber neuropathy and symptom inventory questionnaire

^a^In the authors’ clinical experience

^b^These scales are non-linear so the impact of a specific score change may differ according to the patient’s starting level

**Table 4:** Assessments for monitoring progression in cardiac dysfunction

Technique	Indicator of progression	Frequency of assessment	Sensitivity to progression^a^
Clinical examination	Progression indicated by:	3–6 months	High
	New signs and symptoms of CHF		
	Unplanned cardiac hospitalization		
	Uncontrolled heart failure that would request to increase the diuretic dosage or the need of using intravenous diuretics		
6MWT	If no disabling neuropathy, progression indicated by a decrease of 20–30 m	6 months	High
	Check heart rate response during 6MWT for chronotropic incompetence		
12-lead ECG	New bundle branch block or AV block of any degree	6 months	High/medium
	New microvoltage or pseudo myocardial infarction pattern; new arrhythmias (atrial and ventricular, atrial fibrillation, bradycardia, AV block)		
Holter ECG	New arrhythmias, burden of atrial fibrillation, need for pacing, VT/VF. If new syncope: repeat Holter for sinus dysfunction, atrial fibrillation, atrial or ventricular arrhythmias, and consider EPS	1 year	High
EPS	Asymptomatic conduction abnormalities (left or right bundle branch block and/or prolonged PR interval)	When clinically indicated based on clinical or ECG changes	Medium
	New conduction abnormalities or indication for pacemaker or defibrillator implantation according to existing guidelines or clinical situation		
Pacemaker memory	Check for bouts of asymptomatic atrial fibrillation requiring anticoagulation	6 months	Medium
	Check for worsening of AV block degree if device has a function for preservation of physiologic AV conduction information		
Echocardiography^b^	Myocardial thickness and regional LV strain measurement mandatory. Doppler filling parameters, EF. Strain measurements	1 year	Medium
	Progression indicated by:		
	Increased myocardial thickness (wall thickness 2 mm increase with other symptoms/findings)		
	Decreased basal strain		
	Worsening diastolic dysfunction		
	Decrease in EF		
	Same operator should be used for consecutive assessments		
Cardiac magnetic resonance imaging^b^	Changes noted in the report; T1, ECV, wall thickness, EF	1 year when clinically indicated by ambiguous echo changes	High
Scintigraphy with bone tracers^b^	PYP or DPD cardiac uptake using qualitative Perugini grading 1–3, quantification using H/L ratio. Repeat scan only if initial scan was negative, and if > 3 years, and echo shows significant increase in wall thickness	3 years	High
	Do not repeat once scan is positive		
Cardiac staging system [70, 78]	Persistent change in the patients’ Grogan or Gillmore stage	6 months	Medium
Cardiac biomarkers: NT-proBNP, troponin I, troponin T	Progression indicated by trend increase	3–6 months	High

*6MWT* 6-min walk test, *AV* atrioventricular, *CHF* chronic heart failure, *DPD* ^99m^Tc-3,3-diphosphono-1,2-propanodicarboxylicacid, *ECG* electrocardiogram, *ECV* extracellular volume, *EF* ejection fraction, *EPS* electrophysiologic study, *H/L* heart-to-lung, *LV* left ventricular, *NT-proBNP N*-terminal prohormone of brain-type natriuretic peptide, *PYP* ^99m^Tc-pyrophosphate, *T1* longitudinal relaxation time, *VF* ventricular fibrillation, *VT* ventricular tachycardia

^a^In the authors’ clinical experience

^b^Cardiac imaging should be performed at different visits by the same operator or radiologist, on the same machine, using the same software

**Table 5:** Scenarios of clinically significant worsening in ATTRv amyloidosis that may prompt a change of therapy

Area of impairment	Consultant interview/other notable features	Specific questionnaire^a^	Objective marker in consultation	Investigations
Somatic and autonomic neuropathy	1. Extension of paresthesia, pain on the body from lower limbs to the hands		Extension of sensory loss on the body	NCS
				SNAP amplitudes
	2. Worsening of disability and development of upper limb weakness in previous sensory polyneuropathy	R-ODS	Reduction by 3–8 kg of grip strength	Jamar both hands grip strength test
			Increase by 7–16 points of NIS	NIS
	3. Onset or worsening of gait and/or balance disorders	Reduction of walking perimeter	Increase by 20% for gait speed	10MWT
			Extension of vibration loss in lower limbs	Timed Get Up and Go test
		PND score		Romberg sign/pallesthesia in LL
		Onset of falls		NIS
	4. Onset of erectile dysfunction, diarrhea, orthostatic faintness, urinary disorders, syncope			
	5. Worsening of autonomic manifestation (OH, GI, GU)	CADT	OH onset	MIBG scintigraphy
		COMPASS-31	Sudoscan^®^	HRV testing
Amyloid cardiomyopathy	1. Worsening dyspnea, weight gain, and other symptoms		Echocardiogram parameters	Echocardiogram
			Change in prescription	Biomarkers
			NT-proBNP	
	2. New ECG features		Microvoltage	12-lead ECG
			Atrial fibrillation	Holter ECG
			Conduction abnormalities	EPS
	3. Worsening arrhythmic burden		Pacemaker implantation	Echocardiogram
			Bundle branch block	Biomarkers
			Atrioventricular block	EPS
	4. Worsening echocardiogram parameters confirmed by cMRI		Increased wall thickness	Echocardiogram
			Elevated LV filling pressures	cMRI
			Elevated pulmonary artery pressures	
			New LV dysfunction	
			Worsening of the longitudinal global strain	
	5. Worsening cMRI T1 and ECV measurements			cMRI
				Complete cardiac investigation, DPD scintigraphy
	6. Unplanned hospitalization		Hospitalization	Complete cardiac investigation
General health	1. Weight loss		Weight	mBMI
	2. Well-being	Norfolk QOL-DN		
		NYHA class		

*10MWT* 10-m walk test, *ATTRv* hereditary transthyretin (v for variant), *CADT* compound autonomic dysfunction test, *cMRI* cardiac magnetic resonance imaging, *COMPASS-31* Composite Autonomic Symptom Score-31, *DPD* ^99m^Tc-3,3-diphosphono-1,2-propanodicarboxylicacid, *ECG* electrocardiogram, *ECV* extracellular volume, *EPS* electrophysiologic study, *GI* gastrointestinal, *GU* genitourinary, *HRV* heart rate variability, *LL* lower limbs, *LV* left ventricular, *mBMI* modified body mass index, *MIBG* metaiodobenzylguanidine, *NCS* nerve conduction study, *NIS* neuropathy impairment score, *Norfolk QOL-DN* Norfolk Quality of Life-Diabetic Neuropathy, *NT-proBNP N*-terminal prohormone of brain-type natriuretic peptide, *NYHA* New York Heart Association, *OH* orthostatic hypotension, *PND* polyneuropathy disability, *R-ODS* Rasch-built Overall Disability Scale, *SNAP* sensory nerve action potential, *T1* longitudinal relaxation time

^a^Questionnaires should be filled in before each 6-month consultation

Progression should also be viewed in light of the aggressive nature of ATTRv amyloidosis, such that rate of worsening remains an important measure for physicians.

### Somatic neuropathy

Somatic neuropathy in ATTRv amyloidosis frequently affects the distal lower limbs first, followed by the distal upper limbs, with the neuropathy spreading proximally as the disease advances. However, the upper limbs are also affected earlier in the course of ATTRv amyloidosis than other neuropathies.

The appearance of new somatic neuropathy symptoms or the worsening of existing signs or symptoms may indicate progression (Tables 2, 3).

#### Gait disturbances

In general, FAP stage is too insensitive to be useful in tracking gradual disease progression, especially in patients with EO V30M, as it may take 5 years to transition between stages [56]. However, changes in PND score can occur approximately every 18 months in patients with LO V30M disease [44, 45, 56], signposting increased functional impairment and thus progression.

#### Disability

Alternative measures of neuropathy impairment include the R-ODS score; here changes of −4.0 points and −8.9 points over 9 and 18 months, respectively, were observed in the placebo arm of the APOLLO trial in patients with ATTRv amyloidosis with polyneuropathy (from a baseline mean [SD] of 29.8 [10.8]) [32]. As such, this can be used as a guide as to whether the patient’s disease is progressing [86], although this subjective score should be assessed in association with objective tests.

### Autonomic neuropathy

A case for disease progression can be made if a current autonomic symptom worsens or new symptoms develop. For example, the combination of de novo persistent diarrhea with weight loss could indicate progression. Alternatively, the onset of orthostatic hypotension may herald disease progression. Table 3 includes a list of tests that have been used to detect autonomic symptoms and their sensitivity to progression.

### Amyloid cardiomyopathy

In contrast to the symptoms of neuropathy, some cardiac symptoms do not worsen in a linear fashion but rather follow a threshold pattern or may be reversible over the short to medium term with suitable symptomatic treatment, such as diuretics, despite underlying progression of cardiac impairment. This situation complicates the use of worsening cardiac symptoms or NYHA classification (Table 2) alone to evaluate the progression of cardiac dysfunction, yet a range of potential avenues of investigation are available (Table 4).

The progression of amyloid cardiomyopathy should be suspected if the clinical events described in Table 5 are observed. However, care must be taken during the interpretation of these assessments and certain caveats should be considered. While the combination or worsening of echocardiogram parameters and cMRI parameters can indicate progression, both modalities have less than perfect reproducibility and problems detecting meaningful short-term variations. For example, in the 30-month ATTR-ACT study, the treatment difference in the interventricular wall thickness determined by echocardiogram was less than 0.5 mm and not reported as being significant, despite significant improvements in other disease measures (e.g. 6MWT and KCCQ-Overall Summary score) [84]. However, instead of absolute size measurements, recent data suggest that changes in echocardiographic strain measurements may serve as a more specific measure of progression in cardiac amyloidosis [87].

Among the other imaging methodologies, cMRI T1 and extracellular volume measurements have high sensitivity which allow changes to be observed between consecutive scans. However, caution should be used as these measurements have a wide standard deviation. With respect to scintigraphy, this technique represents a major advancement for diagnosis, but it lacks spatial resolution and its ability to provide absolute quantification of amyloid load remains to be demonstrated, and thus cannot be used widely to accurately assess modest variations during longitudinal follow-up. Furthermore, and considering radiation exposure, scintigraphy should only be repeated if the initial scan was negative, and then in the case of suspicion of disease progression based on clinical judgment, modifications of ECG, NT-proBNP, and/or echocardiography or cMRI, with a minimal interval of 3 years.

Both cardiac amyloidosis staging systems use levels of biomarkers (high-sensitivity troponin and NT-proBNP) that reflect myocyte injury and stress rather than factors directly driving disease progression [70, 78]. As such, they fail to capture progression in cardiac dysautonomia and conduction abnormalities. Furthermore, NT-proBNP levels can fluctuate in response to atrial fibrillation, diuretic treatment, or renal insufficiency [88] and for this reason some clinicians prefer to use the staging systems as prognostic instruments rather than tools to track progression. Staging assessment can only provide insight into the status of cardiac dysfunction when used in conjunction with other appropriate tests, although a persistent change in the patient’s stage could also be recognized as disease progression.

In summary, clinical judgment should remain the cornerstone of patient assessment when monitoring cardiac disease progression. Clinical examination, ECG, echocardiography (by the same operator), and biomarker analysis should be performed at each appointment. Care must be taken when looking for specific combinations of symptoms, weight, and the need for diuretics dose adjustment. All other modalities should be used as clinically needed.

### General health and other organs

Signs and symptoms to consider for disease progression in other organs and systems are listed in Table 2. Onset of new ophthalmologic dysfunction can be captured by testing for ACVs, KCS, vitreous opacities, pupillary abnormalities, and glaucoma at monitoring visits. eGFR should be monitored carefully as a reduction can indicate progression in renal dysfunction and low values are a predictor of mortality [78]. Onset of albuminuria and urine proteinuria can also indicate disease progression.

#### Weight change

Among the symptoms of general health, weight loss is very important. In combination with worsening or onset of other symptoms, reduction in mBMI of 12% over 18 months (the decline in mBMI observed in patients treated with placebo in the APOLLO trial from a baseline mean [SD] of 989.9 [214.2]) [32] could be an indicator of disease progression.

#### Well-being

There is good evidence from a number of clinical trials that decline in QOL can be used as a parallel, holistic measure of disease progression [32, 33, 48, 84]. As a guide to aid clinical judgment, a change in Norfolk QOL-DN of + 14.4 points has been observed over 18 months from a baseline mean (SD) of 55.5 (24.3) in a placebo-treated population of patients with either FAP stage 1 (48%), or FAP stage 2 or 3 (51%) disease [32]. Additionally, a change in Norfolk QOL-DN of approximately + 4 points over 12 months from a baseline mean (SD) of 30.8 (26.7) has been observed in patients treated with placebo with V30M FAP stage 1 disease [47]. Similarly, using data from the placebo arm of clinical studies, a change in the SF-36 physical component of − 1.9 points was observed over 12 months from a baseline mean (SD) of 34.8 (11) in patients with ATTRv amyloidosis with polyneuropathy (FAP stage 1 [66.6%], or FAP 2 or 3 [33.4%]) [48], while the KCCQ score decreased by approximately − 5.6 points over 6 months from a baseline mean (SD) of 65.9 (21.7) in patients with ATTRv amyloidosis with cardiomyopathy at NYHA ≤ III [84]. However, QOL questionnaires are subjective measurements and should always be assessed in association with objective tests.

## Frequency of monitoring and time required to confirm worsening

**Fig. 1:**
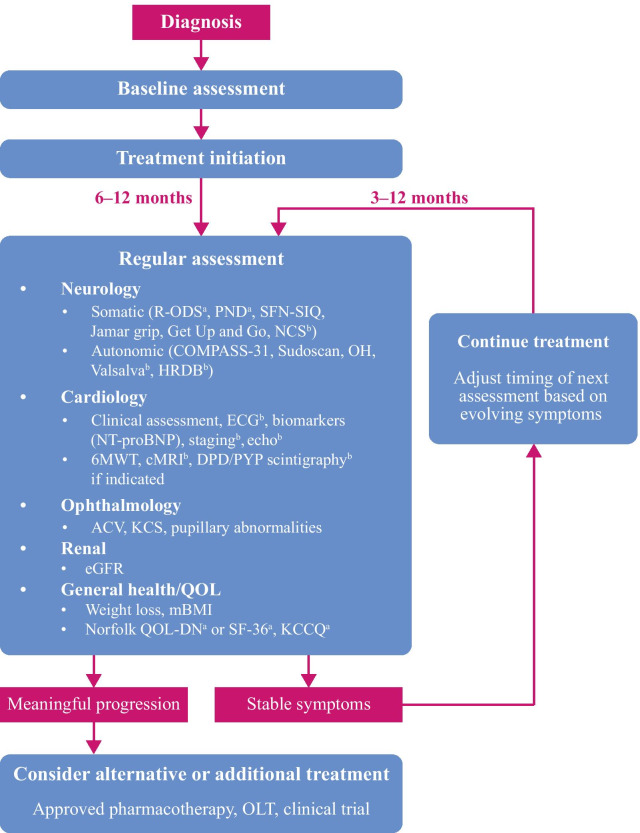
Disease-monitoring algorithm. ^a^Questionnaire to be performed prior to consultation. ^b^Additional test. *6MWT* 6-min walk test, *ACV* abnormal conjunctival vessel, *cMRI* cardiac magnetic resonance imaging, *COMPASS-31* Composite Autonomic Symptom Score-31, *DPD* ^99m^Tc-3,3-diphosphono-1,2-propanodicarboxylicacid, *ECG* electrocardiogram, *eGFR* estimated glomerular filtration rate, *HRDB* heart rate deep breathing, *KCCQ* Kansas City Cardiac Questionnaire, *KCS* keratoconjunctivitis sicca, *mBMI* modified body mass index, *NCS* nerve conduction study, *Norfolk QOL-DN* Norfolk Quality of Life-Diabetic Neuropathy, *NT-proBNP N*-terminal prohormone of brain-type natriuretic peptide, *OH* orthostatic hypotension, *OLT* orthotopic liver transplantation, *PND* polyneuropathy disability, *PYP* ^99m^Tc-pyrophosphate, *QOL* quality of life, *R-ODS* Rasch-built Overall Disability Scale, *SF-36* 36-item Short-Form Healthy Survey, *SFN-SIQ* small-fiber neuropathy and symptom inventory questionnaire

Predicting the symptoms and rapidity of progression from the patient’s *TTR* genotype is difficult due to the variability of ATTRv amyloidosis. Therefore, even patients presenting with only 1 class of symptoms (e.g. neuropathy or cardiac) should have at least a yearly follow-up with appropriate specialists to check the different classes of ATTRv amyloidosis symptoms (somatic and autonomic neuropathy, amyloid cardiomyopathy, and other disease manifestations). Figure 1 summarizes monitoring of ATTRv amyloidosis in a simple algorithm covering baseline assessment, treatment initiation, and follow-up.

Assessments can be adjusted based on the patient’s evolving symptoms. For example, stable patients (e.g. post-orthotopic liver transplantation patients with EO V30M disease) may only need to be seen once a year whereas the frequency of monitoring should be increased to once every 6 months in patients with worsening neurologic scores and symptoms. For patients with predominant peripheral neuropathy, monitoring over at least 1 year, including 2 consecutive measures, is needed to confirm worsening.

In patients with cardiac involvement, the typical interval should be 6 months adjusted by clinical judgment of risk. For example, the period between assessments could be shortened if the patient shows an increased severity of HF and/or HF events, whereas it could be extended in patients who respond well to symptomatic treatments. For V122I, T60A, and other variants or familial history associated with severe amyloid cardiomyopathies, more aggressive monitoring with cardiac screening is recommended. Patients should also be screened with echocardiography every 12 months, and with cMRI if clinically indicated.

## Treatment initiation

Each patient with symptomatic ATTRv amyloidosis should benefit from DMT, with early treatment imperative as ATTRv amyloidosis is rapidly progressive [1]. While symptomatic treatment is a key consideration for physicians, patients with a diagnosis of ATTRv amyloidosis should be prescribed a DMT immediately, providing that the symptoms can be proven to be related to the disease.

Based on current country-specific indications, clinicians should consider which therapies are suitable for the patient’s symptoms (polyneuropathy, cardiac amyloidosis, or both) and the patient’s disease stage. At present, there are no DMTs for the ocular or central nervous system symptoms of ATTRv amyloidosis, although RNA interference agents targeting these systems are under development [89, 90]. Furthermore, strategies to reduce TTR levels are also in planned/ongoing studies in patients with ATTR amyloidosis with cardiomyopathy [91, 92]. It should also be noted that there is currently a lack of head-to-head evidence for the approved therapies, so no universal guidelines can be proposed, and thus clinical judgment should be exercised over the choice of treatment.

## Conclusions

ATTRv amyloidosis is a rare, progressive, and fatal disease in which early therapeutic intervention is key to achieving better patient outcomes. While recently approved DMTs have greatly enhanced treatment options, they have also highlighted the need for guidance on managing ATTRv amyloidosis. In order to monitor the disease course, clinicians should undertake detailed assessment of the multiple symptoms and signs of somatic and autonomic neuropathy, cardiac dysfunction, and other disease manifestations at baseline and during follow-up. Regular monitoring of signs and symptoms (both patient-based assessments and follow-up by the same physician) across these categories is required to detect disease progression and identify non-responders, supported by clinical scales, additional tests, and biomarkers. This multisystem approach to management reflects the mixed phenotype observed in the majority of symptomatic patients and highlights the need to develop therapies that target disease pathophysiology that can thus impact multiple manifestations.

There have been great advances in treatments for ATTRv amyloidosis. However, the management of patients whose disease has progressed despite first-line therapy is currently uncertain. Clearly, there is a need for agreement on how to identify non-responders to new treatments and how/when to change their treatment. We hope that our recommendations can contribute toward this goal by providing definitions and examples of clinically meaningful disease progression. These proposals should be validated by a wider group of physicians using the Delphi method to reach a consensus on monitoring disease progression in ATTRv amyloidosis. Treatment of asymptomatic patients with proven target organ involvement will be the subject of future guidance.

## Supplementary Information

**Additional file 1.** Survival of patients with ATTRv amyloidosis.

## Data Availability

Not applicable.

## Abbreviations

6MWT: 6-min walk test
10MWT: 10-m walk test
ACV: Abnormal conjunctival vessel
ATTRv: Hereditary transthyretin (v for variant)
AV: Atrioventricular
BMI: Body mass index
BP: Blood pressure
CADT: Compound Autonomic Dysfunction Test
CHF: Chronic heart failure
cMRI: Cardiac magnetic resonance imaging
COMPASS-31: Composite Autonomic Symptom Score-31
DMT: Disease-modifying therapy
DPD: ^99m^Tc-3,3-diphosphono-1,2-propanodicarboxylicacid
ECG: Electrocardiogram
ECV: Extracellular volume
EF: Ejection fraction
eGFR: Estimated glomerular filtration rate
EO: Early-onset
EPS: Electrophysiologic study
FAP: Familial amyloid polyneuropathy
GI: Gastrointestinal
GU: Genitourinary
HF: Heart failure
HRDB: Heart rate deep breathing
H/L: Heart-to-lung
HRV: Heart rate variability
IV: Intravenous
KCCQ: Kansas City Cardiac Questionnaire
KCS: Keratoconjunctivitis sicca
LL: Lower limb
LO: Late-onset
LV: Left ventricular
mBMI: Modified body mass index
MIBG: Metaiodobenzylguanidine
NCS: Nerve conduction study
NIS: Neuropathy impairment score
Norfolk QOL-DN: Norfolk Quality of Life-Diabetic Neuropathy
NT-proBNP: *N*-Terminal prohormone of brain-type natriuretic peptide
NYHA: New York Heart Association
OH: Orthostatic hypotension
OLT: Orthotopic liver transplantation
PND: Polyneuropathy disability
PYP: ^99m^Tc-pyrophosphate
QOL: Quality of life
R-ODS: Rasch-built Overall Disability Scale
SF-36: 36-Item Short-Form Health Survey
SFN-SIQ: Small-fiber neuropathy and symptom inventory questionnaire
SNAP: Sensory nerve action potential
T1: Longitudinal relaxation time
UL: Upper limb
VAS: Visual analog scale
VF: Ventricular fibrillation
VT: Ventricular tachycardia
