# Supplementary material for: Expert opinion on monitoring symptomatic hereditary transthyretin-mediated amyloidosis and assessment of disease progression
Source: Orphanet J Rare Dis. 2021 Oct 3;16:411. doi: 10.1186/s13023-021-01960-9 (PMC8489116; doi:10.1186/s13023-021-01960-9)
Supplement: Supplementary file 1 — Additional file 1. Survival of patients with ATTRv amyloidosis. [file 13023_2021_1960_MOESM1_ESM.docx]

# Adams et al.

Additional information

**Table S1. Survival of patients with ATTRv amyloidosis**

| *TTR* Variant | *n* | Median Survival from Disease Onset (Years) | Median Survival from Diagnosis (Years) | Country | Reference |
| --- | --- | --- | --- | --- | --- |
| V30M (p.V50M) early-onset | 483 | 10.8^a^ | – | Portugal | Coutinho et al. 1980 [1] |
|  | 1,771 | 11.6 | – | Portugal | Coelho et al. 2018 [2] |
|  | 110 | 16.9 | – | Portugal | Mariani et al. 2015 [3] |
| V30M (p.V50M) late-onset | 50 | 7.3^a^ | – | Japan | Koike et al. 2012 [4] |
|  | 37  42 | 7.6  – | _  3.5 | France  US | Mariani et al. 2015 [3]  Swiecicki et al. 2015 [5] |
|  | 37 | – | 5.7^b^ | US | Arruda-Olson et al. 2013 [6] |
| V122I (p.V142I) | 28 | – | 3.0 | US | Givens et al. 2013 [7] |
|  | 11 | – | 2.1 | US | Ruberg et al. 2012 [8] |
|  | 30 | – | 2.3 | US | Connors et al. 2009 [9] |
|  | 28 | – | 2.1 | US | Swiecicki et al. 2015 [10] |
|  | 21 | – | 3.3^b^ | US | Arruda-Olson et al. 2013 [6] |
| T60A (T80A) | 68 | – | 3.2 | US | Swiecicki et al. 2015 [10] |
|  | 60 | 6.6 | 3.4 | UK and Canada | Sattianayagam et al. 2012 [11] |
|  | 58 | – | 3.7^b^ | US | Arruda-Olson et al. 2013 [6] |
| S77Y (S97Y) | 15 | – | 4.0 | US | Swiecicki et al. 2015 [10] |
|  | 32 | 12.5 | – | France | Mariani et al. 2015 [3] |
| I107V (I127V) | 15 | 6.8 | – | France | Mariani et al. 2015 [3] |

^a^Mean survival, instead of median survival. ^b^Estimated from Kaplan–Meier plot [6]

ATTRv, hereditary transthyretin (v for variant); TTR, transthyretin

# References

1. Coutinho P, DeSilva AM, Lima JL, Barbosa AR. Forty years of experience with type I amyloid neuropathy: review of 483 cases. In: Glenner G, Costa P, de Freitas A, editors. Amyloid and Amyloidosis. Amsterdam: Excerpta Medica; 1980. p. 88–98.
2. Coelho T, Inês M, Conceição I, Soares M, de Carvalho M, Costa J. Natural history and survival in stage 1 Val30Met transthyretin familial amyloid polyneuropathy. Neurology. 2018;91(21):e1999–2009.
3. Mariani LL, Lozeron P, Theaudin M, Mincheva Z, Signate A, Ducot B, et al. Genotype-phenotype correlation and course of transthyretin familial amyloid polyneuropathies in France. Ann Neurol. 2015;78(6):901–16.
4. Koike H, Tanaka F, Hashimoto R, Tomita M, Kawagashira Y, Iijima M, et al. Natural history of transthyretin Val30Met familial amyloid polyneuropathy: analysis of late-onset cases from non-endemic areas. J Neurol Neurosurg Psychiatry. 2012;83(2):152–8.
5. Swiecicki PL, Zhen DB, Mauermann ML, Kyle RA, Zeldenrust SR, Grogan M, et al. Hereditary ATTR amyloidosis: a single-institution experience with 266 patients. Amyloid. 2015;22(2):123–31.
6. Arruda-Olson AM, Zeldenrust SR, Dispenzieri A, Gertz MA, Miller FA, Bielinski SJ, et al. Genotype, echocardiography, and survival in familial transthyretin amyloidosis. Amyloid. 2013;20(4):263–8.
7. Givens RC, Russo C, Green P, Maurer MS. Comparison of cardiac amyloidosis due to wild-type and V122I transthyretin in older adults referred to an academic medical center. Aging Health. 2013;9(2):229–35.
8. Ruberg FL, Maurer MS, Judge DP, Zeldenrust S, Skinner M, Kim AY, et al. Prospective evaluation of the morbidity and mortality of wild-type and V122I mutant transthyretin amyloid cardiomyopathy: the Transthyretin Amyloidosis Cardiac Study (TRACS). Am Heart J. 2012;164(2):222–8 e1.
9. Connors LH, Prokaeva T, Lim A, Theberge R, Falk RH, Doros G, et al. Cardiac amyloidosis in African Americans: comparison of clinical and laboratory features of transthyretin V122I amyloidosis and immunoglobulin light chain amyloidosis. Am Heart J. 2009;158(4):607–14.
10. Swiecicki PL, Zhen DB, Mauermann ML, Kyle RA, Zeldenrust SR, Grogan M, et al. Hereditary ATTR amyloidosis: a single-institution experience with 266 patients. Amyloid. 2015;22(2):123–31.
11. Sattianayagam PT, Hahn AF, Whelan CJ, Gibbs SD, Pinney JH, Stangou AJ, et al. Cardiac phenotype and clinical outcome of familial amyloid polyneuropathy associated with transthyretin alanine 60 variant. Eur Heart J. 2012;33(9):1120–7.
